# Whole-Genome Doubling as a source of cancer: how, when, where, and why?

**DOI:** 10.3389/fcell.2023.1209136

**Published:** 2023-06-05

**Authors:** Natalia Sanz-Gómez, María González-Álvarez, Javier De Las Rivas, Guillermo de Cárcer

**Affiliations:** ^1^ Cell Cycle and Cancer Biomarkers Laboratory, Cancer Biology Department, Instituto de Investigaciones Biomédicas “Alberto Sols“. (IIBM) CSIC-UAM, Madrid, Spain; ^2^ Bioinformatics and Functional Genomics Group, Cancer Research Center (CiC-IBMCC), Consejo Superior de Investigaciones Científicas (CSIC), University of Salamanca (USAL), Salamanca, Spain

**Keywords:** whole-genome doubling, oncogene, aneuploidy, polyploidy, chromosomal instability, endoreduplication, mitotic slippage

## Abstract

Chromosome instability is a well-known hallmark of cancer, leading to increased genetic plasticity of tumoral cells, which favors cancer aggressiveness, and poor prognosis. One of the main sources of chromosomal instability are events that lead to a Whole-Genome Duplication (WGD) and the subsequently generated cell polyploidy. In recent years, several studies showed that WGD occurs at the early stages of cell transformation, which allows cells to later become aneuploid, thus leading to cancer progression. On the other hand, other studies convey that polyploidy plays a tumor suppressor role, by inducing cell cycle arrest, cell senescence, apoptosis, and even prompting cell differentiation, depending on the tissue cell type. There is still a gap in understanding how cells that underwent WGD can overcome the deleterious effect on cell fitness and evolve to become tumoral. Some laboratories in the chromosomal instability field recently explored this paradox, finding biomarkers that modulate polyploid cells to become oncogenic. This review brings a historical view of how WGD and polyploidy impact cell fitness and cancer progression, and bring together the last studies that describe the genes helping cells to adapt to polyploidy.

## 1 Whole-Genome Doubling in animal physiology

From the simplest to the most complex organism, proliferation is executed by the activation of the cell cycle machinery, involving duplication of the DNA content and its half segregation to each daughter cell. However, under certain circumstances, a cell can replicate its DNA without completing subsequent mitosis. This alternative cell cycle results in a cell with double DNA content in a process known as Whole-Genome Doubling (WGD). WGD is not a rare event in mammals, and it is a common feature of different cell types such as cardiomyocytes (Mollova et al., 2013), hepatocytes (Gentric and Desdouets, 2014), trophoblasts (Chapman et al., 1972), megakaryocytes (Trakala et al., 2015), mammary gland epithelia (Rios et al., 2016), urothelium (Wang et al., 2018), or squamous epithelia (Sanz-Gómez et al., 2020).

**FIGURE 1:**
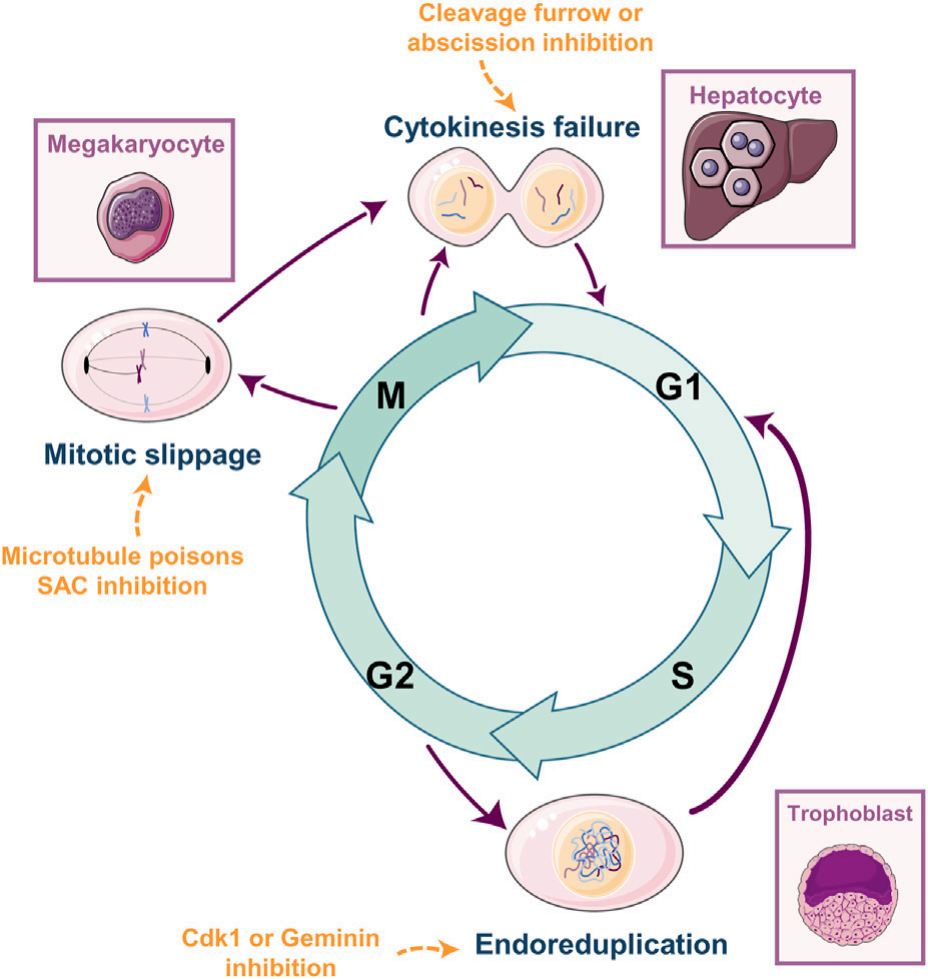
Whole-Genome Doubling: How, and Where. WGD has been described in multiple cell types such as trophoblasts, megakaryocytes, cardiomyocytes, or hepatocytes. The molecular mechanisms leading to WGD are multiple and mainly differ in the specific cell cycle phase from which they arise. Endoreduplication occurs when cells skip entry into mitosis from G2. Mitotic slippage or endomitosis occurs when the cell arrests in mitosis due to activation of the mitotic spindle assembly checkpoint (SAC) and eventually slip mitosis without segregating the genetic material. Cytokinesis failure occurs when cells are unable to properly form the contractile ring at the mitotic midbody and fail to divide. Each event is triggered by different molecular stimuli (examples in orange) and is specific for different cell types and tissues (purple squares). Cartoons were generated using Servier Medical Art, provided by Servier, licensed under a Creative Commons Attribution 3.0 unported license.

A cell undergoing WGD goes from a diploid (2N in the number of chromosomes) to a tetraploid state (4N) (Figure 1) through three main mechanisms: 1) endoreduplication or endocycling, 2) mitotic slippage or endomitosis, or 3) cytokinesis failure. Endoreduplication is the process occurring in trophoblast giant cells upon differentiation (Chapman et al., 1972). This process comprises several rounds of DNA replication (S phase in the cell cycle) without entering mitosis. Endoreduplication is triggered by the inhibition of essential regulators for mitosis entry such as geminin (Gonzalez et al., 2006) or the kinase CDK1 (Ullah et al., 2008). Mitotic slippage was first described in cells that were able to exit mitosis despite being arrested at prometaphase by microtubule poisons such as Nocodazole or Taxol (Andreassen and Margolis, 1994). Megakaryocyte polyploidization, and subsequent maturation, is a typical example of mitotic slippage as a physiological event (Trakala et al., 2015). Mitotic slippage occurs when the cell arrests in mitosis due to activation of the spindle assembly checkpoint (SAC). Active SAC prevents Cyclin B degradation and subsequent CDK1 inhibition, necessary for mitosis exit (Clute and Pines, 1999; Oliveira et al., 2010). Despite an active SAC, a prolonged arrest leads to a slow Cyclin B degradation (Brito and Rieder, 2006) and, when Cyclin B levels fall below a certain threshold, thus inactivating CDK1, the cell resumes mitosis without DNA segregation, generating a tetraploid progeny (Brito and Rieder, 2006; Manchado et al., 2012). Finally, cytokinesis failure is typical of hepatocytes and cardiomyocytes (Margall-Ducos et al., 2007; Hesse et al., 2018; Liu et al., 2019). Cytokinesis failure occurs when cells are unable to properly form the contractile ring at the mitotic midbody, mainly due to chromosome missegregation (reviewed in (Hayashi and Karlseder, 2013)). Alterations in the enzymatic activity or in the expression levels of critical cytokinesis regulators give rise to binucleated cells since karyokinesis is achieved (reviewed in (Normand and King, 2010)). Inhibition of midbody regulators such as Aurora B (Steigemann et al., 2009), Polo-like kinase 1 (PLK1) (Burkard et al., 2007; Petronczki et al., 2007), ECT2 (Chalamalasetty et al., 2006), or overexpression of Aurora A (Meraldi et al., 2002), PLK1 (de Cárcer et al., 2018), or ECT2 (Chalamalasetty et al., 2006), prevents proper furrow formation and cleavage.

Cell fusion is an additional mechanism that induces tetraploidy. Is essential for muscle development or for the formation of multinucleated osteoclasts (reviewed in (Petrany and Millay, 2019)). Cell fusion generates WGD not involving DNA replication or cell cycle progression alterations. A mechanism that leads to pathogenic cell fusion, and subsequent WGD, is a viral infection. This source of WGD is particularly relevant in cancers that are generated by viral vectors such as Hepatitis viruses in liver cancers, Epstein–Barr virus infection in nasopharyngeal tumors, or Human Papilloma Virus (HPV) in anogenital cancers (reviewed in (Duelli and Lazebnik, 2007)). Notably, although viral-dependent WGD is mainly due to cell fusion, these viruses also alter the cell cycle program of infected cells by expressing oncogenes such as E7 and E6. These oncogenes interfere with pRb and p53 signaling, allowing tetraploid cells to proliferate in the absence of these essential checkpoints and ultimately leading to increased replication stress (Yim and Park, 2005).

All the above-described mechanisms lead to cell tetraploidization (4N, number of chromosomes). Tetraploid cells can either rest in the 4N state or undertake further rounds of re-replication increasing proportionally their ploidy (≥4N). In certain tissues, polyploidization fulfills a biological function as part of the developmental program of the cell (Øvrebø and Edgar, 2018; Anatskaya and Vinogradov, 2022). During tissue growth, polyploid cells are more efficient in RNA and protein production and therefore have the ability to increase cell size and biomass production without disrupting cell and tissue structure (reviewed in (Pandit et al., 2013; Øvrebø and Edgar, 2018)). Although recent articles demonstrate that ploidy does not couple linearly with protein levels (Yahya et al., 2022), yet a polyploid cell produces more proteins than a diploid cell does. Consequently, polyploid cells have been described to accomplish specialized functions, for example, an increase in milk production of mammary epithelial cells (Rios et al., 2016).

WGD is classically associated with tissue differentiation and proliferation loss. Additionally, it also has a self-defense anti-proliferative response against damage. For instance, zebrafish epicardial cardiomyocytes become polyploid upon infarction leading to cardiomyocyte hypertrophy ((Cao et al., 2017) and reviewed in (Cao and Poss, 2018)).

In the same trend, the DNA damage leads to WGD as a cellular protective mechanism, in multiple cell types such as hepatocytes (Miyaoka et al., 2012; Gentric et al., 2015), macrophages (Herrtwich et al., 2016), epithelial cells (Pampalona et al., 2012; Sanz-Gomez et al., 2018), and fibroblasts (Davoli et al., 2010).

WGD has also been reported to promote tissue regeneration, for example, in the liver (reviewed in (Sladky et al., 2021)). Some reports show that polyploid hepatocytes have the capacity to regenerate the liver upon injury or hepatectomy (Nevzorova et al., 2009; Diril et al., 2012), Conversely, polyploidization has been described to be detrimental to cardiac (González-Rosa et al., 2018; Han et al., 2020) or hepatic (Wilkinson et al., 2019) regeneration after injury. Reconciling this apparent controversy, it has been shown that these polyploid hepatocytes need to reduce ploidy to a diploid state to recover their proliferative and regenerative capacities (Duncan et al., 2010). Once the liver parenchyma is regenerated,hepatocytes polyploidize again (Matsumoto et al., 2020). In summary, WGD is not a rare event in mammalian tissues. There are different “hows”, “whens”, “wheres” and “whys” that lead to WGD, but one constant is the associated anti-proliferative state. More importantly, the capacity to reverse this lack of proliferation has been shown to have tumorigenic potential.

## 2 WGD in the tumoral context

**FIGURE 2:**
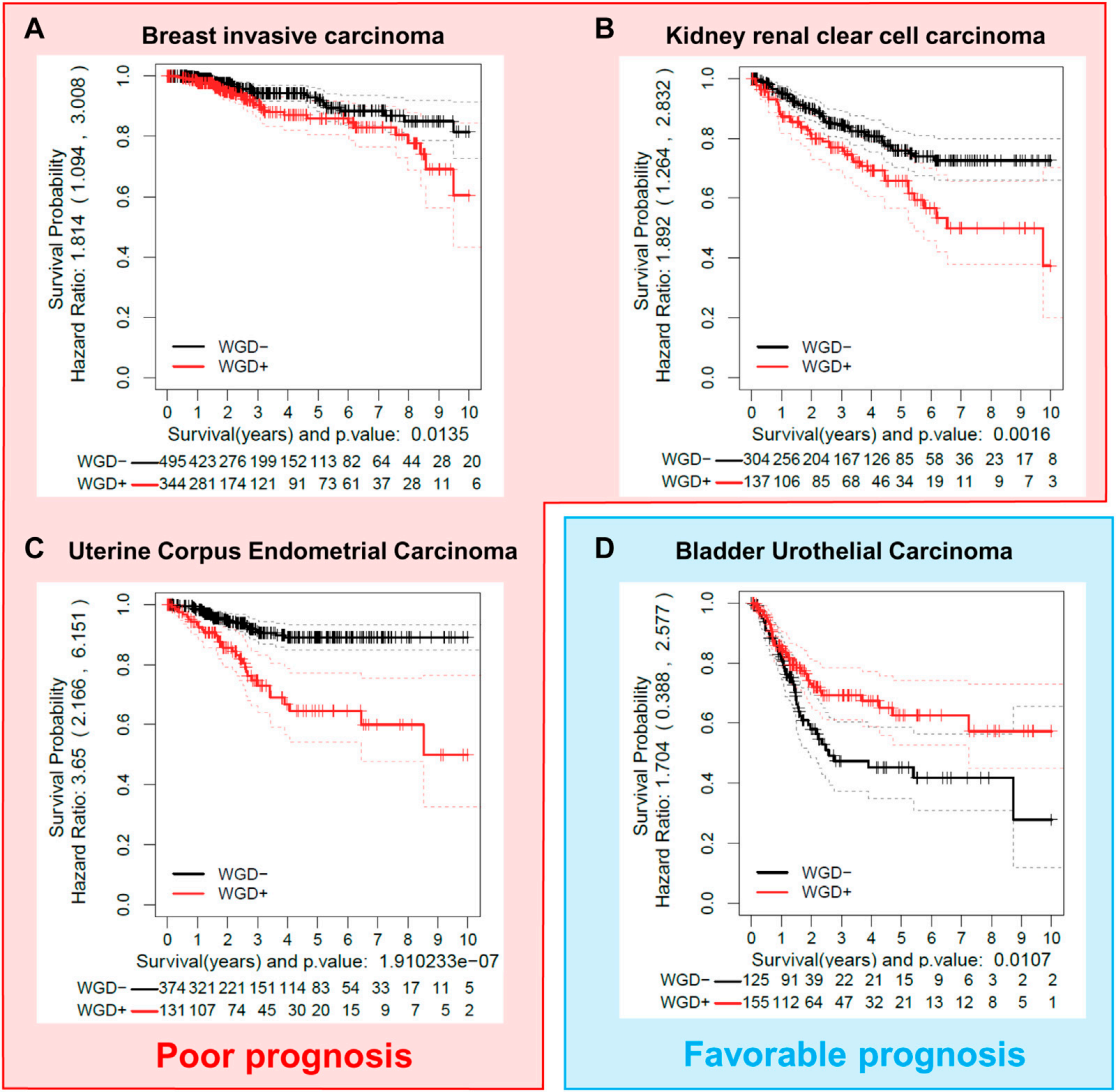
The effect of WGD on tumor prognosis is cancer-type dependent. Different tumor cohorts were examined to show examples of cancer types where WGD provides poor prognosis (**A–C**) (BRCA, Breast invasive Carcinoma; KIRC, Kidney renal clear cell carcinoma; UCEC, Uterine Corpus Endometrial Carcinoma); or favorable prognosis **(D)** (BLCA, Bladder Urothelial Carcinoma), as shown by disease-free specific survival (DSS) in Kaplan-Meier analysis. Data from primary tumor samples were extracted from the TCGA Pan-Cancer Atlas (Weinstein et al., 2013). Ploidy values were obtained from the ABSOLUTE mastercalls file (Carter et al., 2012). WGD groups (WGD- and WGD+) were calculated with the ploidy value and a membership probability estimated by bootstrap (ASURI, R package under preparation (Bueno-Fortes et al., 2023)). Samples with a membership probability greater than 0.8 were considered for the analysis. Kaplan–Meier plots with a fitted Cox model (Therneau, 2023) for the two groups were represented with the Disease Specific Survival (DSS) information of each patient.

WGD has been widely described in multiple types of cancer. Several analyses during the last decade have shown that the presence of WGD is not tissue or tumor type exclusive but it has been found in a vast majority of tumors (Zack et al., 2013; Gerstung et al., 2020; Quinton et al., 2021). The fact that WGD is detrimental to cell proliferation is counterintuitive in the tumoral context since tumoral cells are highly proliferative. Indeed, the impact of WGD on tumor progression is still controversial, and this issue can be shown by simply correlating the disease-specific survival of different tumors with the levels of WGD in each of the tumors analyzed. We have performed a computational analysis using the clinical and ploidy data from the samples available in the TCGA-PanCanAltlas (Weinstein et al., 2013) (Figure 2). This analysis has been performed exclusively in primary tumoral samples, with the aim to eliminate any further genetic alteration of metastatic and more aggressive tumors, that might hinder the survival outcome. Comparing the survival dependency of WGD positive *versus* WGD negative tumors, we find that the WGD impact is tumor-specific. WGD has been considered a bad prognosis factor (showing an oncogenic effect) in many tumor types (Dewhurst et al., 2014; Bielski et al., 2018; Frankell et al., 2023) as we observed for Breast invasive Carcinoma (BRCA), Kidney Renal Clear cell Carcinoma (KIRC), or Uterine Corpus Endometrial Carcinoma (UCEC) (Figures 2A–C). However, in other tumor types such as Bladder Urothelial Carcinoma (BLCA), WGD is beneficial in terms of survival, as shown in the analysis of 280 BLCA tumors presented in Figure 2D. Regarding these data in the BLCA samples, it is important to consider that bladder cancer has some of the highest rates of WGD of any solid tumor and that the rare diploid BLCA tumors appear to have an increased mutational burden compared to the polyploid samples (Quinton et al., 2021), which may confer greater aggressiveness.

Therefore, the next logical question would be: when and how does WGD act promoting tumor progression or suppressing tumorigenesis?

### 2.1 WGD as a tumoral brake

As stated above, WGD is detrimental to cell proliferation, and it is a protective mechanism for cells under stressful circumstances. Even under oncogenic stress, the induction of polyploidy prevents tumor expansion by either blocking cell proliferation, increasing tumor suppressor content, or inducing senescence.

For instance, induction of polyploidy, through PLK1 overexpression, stops cell proliferation and impedes cell transformation mediated by oncogenic K-RasV12. This happens not only *in vitro* in mouse fibroblasts but also in a mouse mammary gland tumoral model expressing oncogenes like H-Ras or Her2 (de Cárcer et al., 2018). PLK1 inducible overexpression leads to WGD by cytokinesis abscission impairment, promoting a strong brake in cell transformation and tumor progression. This data is also observed in human breast tumoral samples, where elevated expression of PLK1, in WGD-categorized tumors, has a better prognostic factor when compared to non-WGD tumors.

Similarly, WGD induction in mouse liver, generated either by early weaning of mouse pups, or by Anillin (*Anln*) knockdown, has a protective effect when mice are later treated with liver carcinogenic agents such as diethylnitrosamine (Zhang et al., 2018). Concomitantly, when the mouse liver is forced to reduce the percentage of polyploid cells, by knocking out *E2f7* or *E2f8* genes (Chen et al., 2012; Pandit et al., 2012), the administration of diethylnitrosamine leads to an increased hepatocyte transformation (Zhang et al., 2018; Wilkinson et al., 2019). E2F7/8 are transcriptional repressors of genes such as ECT2, RacGAP, and MKLP1, which are essential regulators for successful cytokinesis ((Pandit et al., 2012) and reviewed in (Donne et al., 2021)), and their expression is downregulated during hepatocyte binucleation (Margall-Ducos et al., 2007). The explanation for this phenomenon is that tetraploid cells duplicate the copy number of tumor suppressor genes (TSG), therefore being able to buffer its inactivation under the oncogenic stress (López et al., 2020). While in a diploid context, the inactivation of TSG leads to loss of heterozygosity (LOH) and the increasing likelihood of tumorigenesis, the extra TSG copies within a polyploid cell attenuate this probability, thus preventing tumorigenesis (Zhang et al., 2018). In the same trend, WGD induced in the liver by alteration of the PIDDosome complex (either by genetic depletion of *Pidd1* or *Raidd*) or its downstream effector Caspase-2, promotes a protective effect against hepatocarcinoma development (Sladky et al., 2020b). The PIDDosome is a sensor for supernumerary centrosomes, which is a hallmark of tetraploid cells (Fava et al., 2017). Accumulation of extra centrosomes activates the PIDDosome signaling cascade, arresting the cell cycle. Therefore, depletion of the PIDDosome signaling leads to an increase in cellular ploidy (Dorstyn et al., 2012; Sladky et al., 2020a). WGD as tumor protective in the liver has also been shown upon CDK1 inhibition. CDK1 is an essential mitosis entry kinase, and its inhibition leads to endoreduplication or endocycling (Ullah et al., 2008; Diril et al., 2012). Knocking out CDK1 in the liver generates polyploidization while impeding transformation and tumorigenesis in an oncogenic background such as N-RasV12 expression or TP53 depletion (Diril et al., 2012).

Another example of WGD or polyploidy as a tumoral break is nicely represented in skin nevi and melanoma development. Polyploidy is a common feature in benign lesions such as nevi (Isaac et al., 2010; Larijani et al., 2011), which rarely results in malignant melanomas (Tsao et al., 2003; Damsky and Bosenberg, 2017). In fact, healthy melanocytes display WGD under oncogenic conditions such as H-RASV12 (Dikovskaya et al., 2015), N-RAS61K (Leikam et al., 2015), or B-RAFV600E (Darp et al., 2022). This increase in their DNA content is accompanied by senescence in a process known as oncogene-induced senescence (Dhomen et al., 2009). Interestingly, these oncogenic alterations together with the senescent response are also present in nevi (Pollock et al., 2002; Michaloglou et al., 2005). This suggests that the senescence resulting from WGD may prevent premalignant lesions, such as nevi, from progressing to melanoma.

### 2.2 WGD as a tumor inducer

WGD is being robustly demonstrated to be an early event in a wide variety of tumors (Olaharski et al., 2006; Dewhurst et al., 2014; Bielski et al., 2018; Boisselier et al., 2018; López et al., 2020). Indeed, despite being detrimental to cell fitness, WGD has been shown as a starting point to generate chromosomal instability (CIN). Many studies demonstrated that tumoral chromosome copy number alterations (CNAs) predominantly occur after a WGD event, leading to increased levels of CIN (Fujiwara et al., 2005; Ganem et al., 2009; Dewhurst et al., 2014; Thomas et al., 2018; Gerstung et al., 2020; Minussi et al., 2021). A formal demonstration of this WGD-derived CIN, as a tumoral driver, is shown by R. A. Lambuta and colleagues. Here, immortal RPE1 cells subjected to WGD are prone to generate tumors *in vivo* after adapting to the WGD, by suffering severe chromosomal rearrangements while losing the polyploid state leading to CIN. In contrast, control diploid RPE1 cells, not subjected to a WGD event, are unable to generate tumors and do not implement CIN (Lambuta et al., 2023).

Recent studies have demonstrated that chromosomal losses only have tumorigenic potential when they originate after WGD (Thomas et al., 2018). On the other hand, other evolution analyses of tumoral subpopulations have revealed that there is a series of multiple deletions, which occur before polyploidization (Carter et al., 2012; Chan-Seng-Yue et al., 2020; Gerstung et al., 2020; Baslan et al., 2022). For instance, in premalignant pancreatic lesions most mutations and copy number alterations accumulate in the diploid state while the disease is still not invasive (Murphy et al., 2013; Notta et al., 2016). After the preneoplastic period, WGD events are crucial for transformation and tumoral progression (Notta et al., 2016). Therefore, WGD in these tumor progression evolution context acts as a buffer for oncogene amplification. These structural alterations are further reflected in the tumors, as WGD strongly correlates with higher tumoral aneuploidy (Taylor et al., 2018; Vergara et al., 2021; Prasad et al., 2022).

It is important to consider that aneuploidy and CIN can already happen in normal cells. For example, human and murine hepatocytes are often aneuploid and, despite transformation does not necessarily take place, this aneuploidy might generate a genetic diversity background among hepatocytes (Duncan et al., 2012). Noteworthy, the hepatocyte aneuploidy concept is somehow controversial, as others describe that it is not present in murine or human hepatocytes (Knouse et al., 2014). Chromosome-number alterations and aneuploidy have been demonstrated to have no impact or to be detrimental to cell fitness thus preventing tumoral transformation (Weaver et al., 2007; Sheltzer et al., 2017). However, under specific circumstances such as hypoxia or chemotherapy exposure, aneuploidy gives an evolutionary advantage that permits adaptation and eventually survival and further proliferation (Sheltzer et al., 2017; Simões-Sousa et al., 2018). Other authors also demonstrate that contrary to *in vitro* conditions, in the stressful conditions of anchorage-independent growth, aneuploidy gives an advantage towards *in vivo* tumor formation (Thomas et al., 2018). Therefore, aneuploidy provides genetic plasticity which results in adaptative capacity, metastasis, and resistance (Chen et al., 2015; Yates et al., 2015).

**FIGURE 3:**
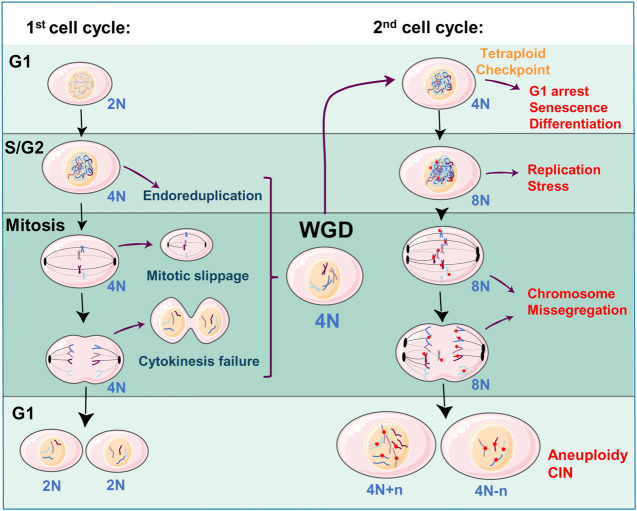
WGD as a source of aneuploidy and CIN: Diploid cells (2N—where ‘N' denotes the number of chromosomes) can slip cell division, during the S/G2 or mitosis phases, leading to a WGD event and a tetraploid state (4N). Tetraploid cells arrest in the next G1 phase due to the activation of a tetraploid checkpoint eventually triggering a non-proliferative state (senescence, terminal differentiation). When 4N cells re-enter the second cell cycle and replicate the DNA (8N), will be exposed to replication stress and chromosome missegregation alterations (DNA damage by red stars, centrosomes by black ovals). Adaptation to these alterations will generate aneuploidy (4N + *n*, 4N-n, where “n” refers to the number of missegregated chromosomes) and further chromosomal instability (CIN). The Figure was partly generated using Servier Medical Art, provided by Servier, licensed under a Creative Commons Attribution 3.0 unported license.

One elegant explanation for the acquired aneuploidy, after a WGD event, is the fact that tetraploid cells undergo replicative stress in the following cell cycle round after the segregation failure. Replication stress is generated by the slowing or stalling of replication fork progression during DNA synthesis. This complex phenomenon has serious implications for genome stability, cell survival, and human disease (reviewed in (Zeman and Cimprich, 2013; Macheret and Halazonetis, 2015)). In addition, it is well known that replication stress is closely related to CIN being both a source and a consequence of CIN (reviewed in (Wilhelm et al., 2020). If a tetraploid cell is able to overcome the G1 arrest, it might prematurely enter the next S phase with insufficient DNA replication factors, generating replicative stress (Gemble et al., 2022). This replicative stress leads to further problems in the cell cycle progression and might generate abnormal karyotypes of over-replicated and down-replicated regions further leading to instability (Figure 3). These initial alterations in the tetraploid cells promote a “post-tetraploid aneuploid” stage where cells increase the rate of chromosome missegregation and therefore increase CIN levels, acquiring advantageous survival opportunities (Viganó et al., 2018). Another explanation for the subsequent aneuploidy and CIN is the existence of supernumerary centrosomes, derived from a WGD event (Figure 3). The presence of supernumerary centrosomes is a hallmark of aggressive tumors (revised in (Godinho and Pellman, 2014)), and it has been shown to promote tumorigenesis (De Cárcer and Malumbres, 2014; Levine et al., 2017). Although it has been proposed that tetraploid cells tend to lose the extra number of centrosomes to avoid further mitotic problems (Baudoin et al., 2020; Galofré et al., 2020) this process of centrosome reduction takes several cell cycle rounds, time enough to generate chromosome missegregation and basal rates of aneuploidy (Figure 3) (Krämer et al., 2003; Ganem et al., 2009).

An alternative reason why WGD leads to CIN is the fact that tetraploid cells have the ability to reduce polyploidy. In the particular case of the liver hepatocytes, the phenomenon of ploidy reduction is referred to as a “ploidy conveyor” (Duncan et al., 2010; Matsumoto et al., 2020). This is a process that occurs during liver regeneration, to facilitate proliferation (Matsumoto et al., 2020), and can lead to chromosome missegregation errors and CIN (Duncan et al., 2010; Matsumoto et al., 2021). In this case, despite WGD in the liver has been proven to be protective, the reduction in ploidy causes the loss of the protective shield of the tetraploidy, which furthermore increases the rate of CIN (Matsumoto et al., 2021).

Once tetraploid cells start chromosome missegregation, adaptation to the acquired CIN is crucial for cell fitness. Whereas high chromosome missegregation frequently generates nullisomy, which is detrimental to cell viability (Janssen et al., 2009; Silk et al., 2013), low rates of missegregation are not sufficient to increase karyotypic heterogeneity, hampering adaptability. Thus, a CIN “sweet spot” occurs when cells find a missegregation rate allowing karyotypic combinations favorable for cell fitness (reviewed in (Bakhoum and Landau, 2017)). These combinations tend to maximize the copies of oncogenic chromosomes and minimize those with a tumor-suppressive disposition (Laughney et al., 2015).

## 3 Genetic determinants modulating the WGD oncogenic-suppressor balance

**FIGURE 4:**
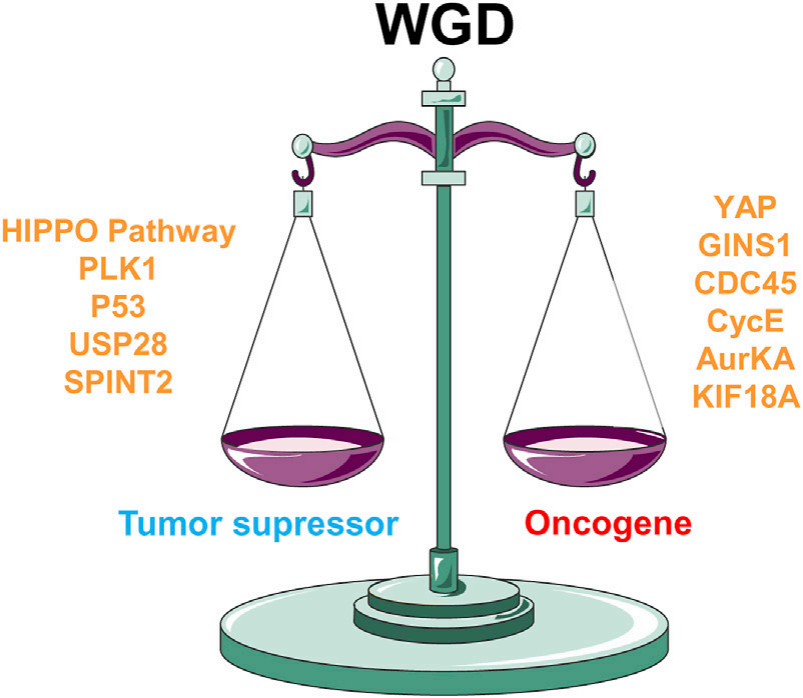
Genetic determinants balancing WGD towards oncogene or tumor suppressor fate. HIPPO signaling, cell cycle genes like PLK1, or DNA damage-related genes such as TP53, USP28, or SPINT2 play as tumor suppressors in a WGD context. On the other hand, increased YAP transcription activity, and overexpression of Cyclin E, Aurora kinase A (AurKA), GINS1, CDC45 or KIF18A leads to oncogenic progression in WGD cells.

WGD *per se* is detrimental to cell viability however, WGD has been found in most tumor types (Quinton et al., 2021). Therefore, the immediate question is which genetic determinants allow WGD to switch from a tumor-suppressor to an oncogenic function. Indeed, it has been shown that WGD-positive tumors converge on genetic alterations leading to an inefficient G1 arrest facilitating cell proliferation (Bielski et al., 2018). We will cluster the possible candidates into two major groups: 1) Cell cycle, DNA damage, and repair pathways and 2) HIPPO-YAP pathway (Figure 4). Tetraploid cells, generated after WGD events, are typically arrested in the subsequent G1 thanks to the activation of the p53 signaling (reviewed in (Aylon and Oren, 2011)) and the HIPPO pathway (Ganem et al., 2014). P53 activation after WGD prevents cells from re-entering the cell cycle and leads to senescence (Notterman et al., 1998; Andreassen et al., 2001; Aylon and Oren, 2011). Furthermore, both pathways, p53, and HIPPO, are interconnected via LATS2 (a HIPPO effector) ensuring the G1 arrest after WGD (Aylon et al., 2006). Even the presence of oncogenic alterations such as B-RAFV600E display WGD (Darp et al., 2022) and activates p53 and HIPPO pathways thus preventing proliferation (Vittoria et al., 2022). In fact, p53, LATS2, and the PIDDdosome have been proposed to form part of a “tetraploid checkpoint” that prevents tetraploid cells from re-entering the cell cycle, since the inactivation of either gene reverses the G1 arrest (Aylon et al., 2006; Aylon and Oren, 2011; Ganem et al., 2014; Sladky et al., 2020b).

### 3.1 Cell cycle, DNA damage, and DNA repair-related signaling

P53 is known as the guardian of the ploidy (Aylon and Oren, 2011). WGD and TP53 loss have been known to be closely linked for decades, with increased WGD specifically associated with p53 loss of function (reviewed in (Aylon and Oren, 2011; Kneissig et al., 2019)). It prevents tetraploid cells to reenter the cell cycle by arresting cells in the next G1 phase and eventually triggers a non-proliferative state (Cross et al., 1995; Andreassen et al., 2001). To overcome the tetraploid barrier, WGD cells need to either inactivate or gain the function of certain signaling pathways, to be able to proliferate and lead to tumoral progression. Downregulation of p53 facilitates re-entrance to the cell cycle (Cross et al., 1995). Indeed, a *Trp53* deficient genetic background helps to avoid the tetraploid checkpoint and facilitates WGD cells to further progress in the cell cycle and promote cell transformation in xenograft models (Fujiwara et al., 2005). Moreover, TP53 deletion also facilitates the generation and perpetuation of haploid cells (Olbrich et al., 2017) demonstrating that p53 is a master regulator of cell ploidy in general. Overall, p53 inactivation has been associated with elevated tolerance to aneuploidy and CIN (Aylon and Oren, 2011; Dorstyn et al., 2012). Evolutional analysis has revealed that the loss of TP53 is an early event before WGD (Gerstung et al., 2020; Baslan et al., 2022). On the other hand, recent studies also demonstrate that p53 needs to be active to generate WGD after replication stress (Zeng et al., 2023), and this is performed by the activation of p21 and promoting a subsequent mitotic bypass by inhibiting cyclin-dependent kinase. Overall, there are many roads, both p53-dependent, and p53-independent, that lead to WGD and tumorigenesis*.* In the particular case of the liver, hepatocytes have been reported to reduce ploidy during liver regeneration to increase proliferation (Matsumoto et al., 2020). P53 participates in this process since TP53 null cells are unable to reduce ploidy even under regenerative pressure. Indeed, TP53 loss induces increasing rates of ploidy together with mitotic defects (Kurinna et al., 2013).

Overexpression of the mitotic regulator Aurora kinase A generates WGD in the mammary gland, in a *Trp53* null background, leading to centrosome duplication and increased genetic instability, and ultimately to tumorigenesis (Wang et al., 2006). However, this is not always the case for mitotic regulator kinases, and the loss of p53 function seems not to be sufficient for cell transformation upon WGD. For example, in mouse fibroblasts (MEFs) where PLK1 overexpression plays as a tumor suppressor, by generating polyploidy, the genetic depletion of *Trp53* does not facilitate cell transformation (de Cárcer et al., 2018). *Trp53* null MEFs overexpressing PLK1 lead to an exacerbated level of polyploidy, when compared to *Trp53* wild-type cells, demonstrating that p53 is a modulator for polyploidy. Despite these MEFs achieving a strong polyploidy status, they cannot be transformed in the presence of oncogenic K-RasV12. Therefore, other determinants besides p53 are needed to allow polyploid cells to transform, and the way WGD and polyploidy are achieved might determine the final tumoral outcome of the altered cells.

Overexpression of cell cycle genes has been shown to have tumoral potential in WGD cells by inducing replication stress and DNA damage. Cyclin E overexpression is well known to induce premature S-phase entry and consequently replication stress. As a result, mitosis progression is prevented and cells undergo polyploidization (Aziz et al., 2019; Lee et al., 2020; Zeng et al., 2023). Overexpression of Cyclin E also triggers missegregation errors and double-strand breaks that are associated with high rates of aneuploidy. Accordingly, mice overexpressing Cyclin E develop spontaneous tumors in the liver with increased nuclear size, indicative of polyploidization (Aziz et al., 2019). A similar effect occurs when essential cell cycle genes for DNA replication, such as GINS1 or CDC45, are overexpressed in chromosomally stable HCT116 cells. Elevated levels of GINS1 or CDC45 cause increased replication origin firing, which interestingly leads to altered microtubule dynamics during mitosis and subsequent chromosome missegregation and eventually WGD (Böhly et al., 2022). Similar data have been described by others showing that the induction of replication stress, by a variety of means, results in whole chromosome missegregation (Greil et al., 2015; Wilhelm et al., 2019).

This is consistent with the fact that tetraploid cells that re-enter the cell cycle have increased replication stress (Gemble et al., 2022), and even the DNA damage response is activated during mitosis of the second cell cycle after WGD (Figure 3). Moreover, after a binucleation event, the cell cycle of both nuclei can be asynchronous which further generates DNA damage in the delayed nucleus (Nano et al., 2019). Consequently, the upregulation of DNA repair-related genes is important for tetraploid cells to escape replication stress-induced senescence (Zheng et al., 2012). In fact, polyploid cells have an increased DNA repair capacity which gives them an advantage against genotoxic agents (Ivanov et al., 2003). Hence, specific genes associated with the DNA damage response have been found to modulate the proliferative capacity of cells undergoing WGD. For example, USP28 activates the DNA damage response upon centrosomal stress. Inhibition of USP28 in the presence of DNA damage alleviates the response and facilitates the proliferation of tetraploid cells (Bernhard et al., 2022).

Another mechanism for generating WGD is caused by critically short telomeres, leading to sustained DNA damage. In this scenario, cells become tetraploid and are arrested in the G1 phase. However, after inhibition of p53, the cells re-enter the S phase and thus acquire a higher tumorigenic potential. The restoration of telomere protection in tetraploid cells, leads to the restart of cell division cycles and proliferation, leading to chromosome loss and adaptation to tetraploidy (Davoli et al., 2010; Davoli & de Lange, 2012). Therefore, the telomere maintenance mechanisms can also modulate adaptation to WGD in a tumoral context.

Since WGD is detrimental to cell viability, it is reasonable to speculate that WGD cells need to relay on certain genetic determinants for their survival. This has been confirmed by *in silico* studies, correlating the cell dependency in genome-wide shRNA screens, and the WGD status. The mitotic kinesin KIF18A recently emerged as an essential gene for WGD-positive cells, conferring unique dependency for elevated CIN cells, whereas is dispensable in normal diploid cells (Cohen-Sharir et al., 2021; Marquis et al., 2021; Quinton et al., 2021). KIF18A is found overexpressed in a wide variety of tumors, and promotes carcinogenesis (Liao et al., 2014; Cohen-Sharir et al., 2021), therefore KIF18A overexpression might provide a window for tetraploidy tolerance and further tumor progression. Concomitantly, KIF18A inhibitors have been proven to be effective as an antitumoral therapeutic strategy in preclinical models (Sabnis, 2020).

In a similar fashion study, by employing a siRNA screen, first the Pellman’s laboratory, and later the Storchova’s laboratory, found that SPINT2 gene downregulation was fundamental for tetraploid cell survival (Ganem et al., 2014; Bernhard et al., 2022). SPINT2 plays as a negative regulator of growth factor signaling, suggesting that persistent growth factor signaling may be needed to overcome the cell cycle arrest upon WGD (Ganem et al., 2014). The proposed mechanism is that SPINT2 is a general regulator of CDKN1A (p21) transcription via histone acetylation, thus positively regulating the expression of p21 (Bernhard et al., 2022). Interestingly, the p21 modulation by SPINT2 is independent of p53 activity. Therefore, SPINT2 downregulation in tetraploid cells enables to bypass G1 arrest, upon various cellular stresses and can provide a proliferative advantage and CIN adaptation, even in the presence of p53.

#### 3.2 HIPPO pathway

The HIPPO signaling pathway is an essential signaling axis in cancer. Mutations in elements of the pathway are found in human cancers, and the same mimicking mutations in the mouse orthologous genes result in tumorigenesis (reviewed in (Saucedo and Edgar, 2007; Harvey et al., 2013)). The HIPPO pathway (consisting mainly of LATS1, LATS2, MST1, and MST2 kinases) is also a major sensor for WGD and tetraploidy, through one of its main players LATS2 that prevents the cell cycle re-entering of WGD cells (Iida et al., 2004; Aylon et al., 2006; Ganem et al., 2014). The HIPPO pathway is a negative regulator of YAP/TAZ activity. YAP/TAZ are transcription factors that induce the expression of genes related to survival, proliferation, migration, and invasion (Totaro et al., 2018). The impact of the HIPPO-YAP signaling axis, during polyploidization, has been extensively studied in megakaryocytes (Roy et al., 2016), hepatocytes (Lee et al., 2016; Zhang et al., 2018), fibroblasts (Mukai et al., 2015), epithelial cells (Losick et al., 2016), cardiomyocytes (Liu et al., 2019) and trophoblast giant cells (Basak and Ain, 2022).

Activation of the YAP/TAZ signaling, by shutting down the HIPPO pathway or direct YAP overexpression, leads to an increase in polyploidization in hepatocytes and MEFs, together with a p53-dependent proliferation arrest. Consequently, HIPPO inactivation in a *Trp53* null background allows polyploid cells to recover their proliferative capacity (Lee et al., 2016; Zhang et al., 2017). Particularly, inhibition of the HIPPO pathway in hepatocytes displays an accumulation of supernumerary centrosomes and abnormal mitosis, eventually leading to genomic instability and tumorigenesis (Zhang et al., 2017). Polyploidy rates increase in a process dependent on Skp2 cytoplasmic retention. Skp2 is a cell cycle regulator whose inhibition leads to polyploidization (Kossatz et al., 2004). Concisely, activation of YAP in hepatocytes activates the Akt-p300 signaling promoting the acetylation of Skp2 that leads to its stabilization and cytoplasmic retention. This signaling results in p27 hyper-accumulation inducing WGD. Interestingly, the inhibition of Skp2 negative effectors (Akt or p27) reduces cell polyploidy and impedes the tumorigenesis induced by HIPPO pathway inhibition (Zhang et al., 2017).

The HIPPO pathway is also a sensor of extra centrosomes. As commented above, tetraploidy leads to an increased number of centrosomes. The presence of supernumerary centrosomes in RPE1 cells, upon a WGD event, results in RhoA GTPase activity inhibition and Rac1 activation. This alteration of the cytoskeleton GTPases RhoA and Rac1 enables HIPPO pathway activity as a safeguard mechanism (Yu et al., 2012; Ganem et al., 2014). The inactivation of this safeguard mechanism is important for megakaryocytes, where RhoA is uncoupled from the HIPPO pathway to permit polyploidy and megakaryocyte maturation for platelet shedding (Roy et al., 2016). Likewise, genetic depletion in MEFs of LATS2 (McPherson et al., 2004; Yabuta et al., 2007) or LATS1 (Yabuta et al., 2013; Mukai et al., 2015) display centrosome amplification, polyploidization, and micronuclei formation as a readout for genomic instability. The proposed mechanism is the binding of LATS1/2 to the centrosome duplication regulator Cdc25B. This binding leads to Cdc25B destabilization, impeding centrosome overduplication (Mukai et al., 2015).

HIPPO signaling is also modulated by precise oncogenic signals, in order to permit cell proliferation in polyploid cells. As mentioned earlier in this article, oncogenic insults such as B-RAFV600E display WGD (Darp et al., 2022). Interestingly, oncogenic B-RAFV600E also induces HIPPO pathway activation and cell cycle arrest *in vitro* as well as in nevus melanocytes *in vivo*. Deletion of HIPPO regulators, such as LATS1/2 in this oncogenic background, has a strong tumorigenic potential permitting oncogenic B-RAF expressing melanocytes to bypass nevus formation (Vittoria et al., 2022). Similarly, the oncogene v-Src is a negative modulator of the HIPPO pathway, by LATS1/2 inactivation, leading to YAP activation and the generation of tetraploid cells (Kakae et al., 2017). Moreover, v-Scr activity has been proposed to cause multipolar mitotic spindles and chromosome missegregation leading to polyploidy (Ikeuchi et al., 2016). Therefore, v-Src plays as an inhibitor of the tetraploid checkpoint, via the HIPPO pathway, permitting the malignant progression of polyploid cells (Nakayama et al., 2017).

In summary, the inactivation of the HIPPO pathway, leading to a YAP transcriptional signaling activation, represents an important adaptive cue for WGD and polyploidy. Indeed, an extensive analysis of a wide variety of cancer cell lines has revealed that high polyploid cancer cells harbor increasing rates of YAP amplification when compared to diploid cells (Ganem et al., 2014).

## 4 Discussion

WGD is a hallmark of cancer (Zack et al., 2013; Bielski et al., 2018; Gerstung et al., 2020; Quinton et al., 2021), and has been widely demonstrated to be an early event in cancer, favoring tumor development (Dewhurst et al., 2014; Boisselier et al., 2018; López et al., 2020). On the other hand, WGD is detrimental to cell fitness and proliferation and is a protective mechanism to evade severe cellular damage (reviewed in (Hayashi and Karlseder, 2013; Gandarillas et al., 2018)). This dichotomy is probably tissue and cell-dependent (Donne et al., 2021), and the balance between the tumor suppressor and oncogenic function is directed by different signaling pathways.

WGD causes missegregation errors and chromosomal instability (CIN), and this is the major mechanism proposed by which WGD can be tumor prone. Nonetheless, CIN also exemplifies the tumor-suppressor vs oncogene paradox. While high rates of CIN lead to cell death and tumor suppression, moderate instability generates heterogenic karyotypes that might be beneficial for cell fitness (Silk et al., 2013; Bakhoum and Landau, 2017). The key to balancing CIN levels and finding the “sweet spot” for tumor development, might reside in the level of DNA damage generated and the capacity of cells to repair it (Ivanov et al., 2003; Weaver et al., 2007; Zheng et al., 2012). Indeed, pure tetraploid cells are inefficient in terms of proliferation and survival and need to lose chromosome dosage to become “near-tetraploid” cells, finding the capacity to survive and acquire the genetic plasticity to be adapted to any external challenge (Taylor et al., 2018; Viganó et al., 2018). In an extreme context, a cell that underwent WGD can completely revert the tetratploid state and become diploid again, but with severe chromosomal rearrangements and CIN that provides transformation capacity (Lambuta et al., 2023).

Another apparent discrepancy is the evolution order of the events related to copy number variation. Different independent analyses have shown that WGD is an early event in tumor evolution leading to chromosome copy number alterations (CNA), aneuploidy, and CIN (Dewhurst et al., 2014; Thomas et al., 2018; Gerstung et al., 2020; Minussi et al., 2021). On the other hand, reports are showing that LOH, CNA, or CIN might accumulate before WGD in the preneoplasic lesions (Murphy et al., 2013; Notta et al., 2016). This might explain why WGD is induced upon these insults to inhibit cell proliferation and protect the cells from further aberrations. Paradoxically, these “defensive” WGD events can provide a scenario to better tolerate missegregation leading to increased instability, eventually favoring major genetic alterations, cell transformation, and increased aggressiveness (Dewhurst et al., 2014).

In summary, when and how WGD leads to a tumoral suppressor or an oncogenic episode is still an unresolved question. Therefore, understanding the genetic determinants that balance either phenotype will bring promising prognostic biomarkers, and eventually can provide new therapeutic opportunities. Up to now, the p53 and HIPPO pathways are the major modulators for this balance. In addition, an increased capacity to recover from the intrinsic DNA replication stress, and subsequent DNA damage, is a major mechanism to overcome the deleterious effect of WGD. Yet there are still open questions in this regard, for example, TP53 depletion seems not to be sufficient for overcoming polyploid tumoral suppression (de Cárcer et al., 2018), despite many other examples proving this mechanism (Meraldi et al., 2002; Fujiwara et al., 2005).

Therefore, further research is needed to find new genetic determinants that can provide mechanisms for WGD adaptation and tumor progression.
